# Admission screening form and osteoporosis educational appointment: a novel fracture liaison service system for identifying osteoporosis patients and facilitating medication initiation

**DOI:** 10.1007/s11657-023-01326-7

**Published:** 2023-09-13

**Authors:** Shunsuke Kikuchi, Yasunori Suda

**Affiliations:** 1 Department of Orthopedic Surgery, International University of Health and Welfare, Shioya Hospital, Yaita City, Tochigi Japan; 2 Department of Orthopedic Surgery, School of Medicine, International University of Health and Welfare, Narita City, Chiba Japan

**Keywords:** Fracture liaison service, Osteoporosis, Screening form, Patient education, Patient identification, Medication initiation

## Abstract

***Summary*:**

Our FLS team aimed to ensure that patients admitted to the orthopedic department were promptly initiated for medication and identify and initiate medication for patients admitted to other departments. Our innovative FLS system along with admission screening and osteoporosis education have proven effective in identifying patients with osteoporosis and initiating medication.

**Purpose:**

The fracture liaison service (FLS) plays a crucial role in the secondary prevention of fragility fractures by involving various medical professionals. Our FLS team had two goals for preventing primary and secondary fractures: ensuring that patients admitted to the orthopedic department were promptly initiated on medication and identifying and initiating medication for patients admitted to other departments.

**Methods:**

From April 2020 to March 2023, we analyzed the number of dual-energy X-ray absorptiometry (DEXA) scans performed, the DEXA rate among patients with proximal femoral fractures, and the rate of medication initiation each year. Our hospital implemented the FLS system in April 2022. It is a unique system utilizing admission screening form and osteoporosis educational appointments conducted by rehabilitation staff to initiate medication for orthopedic and non-orthopedic patients.

**Results:**

The average monthly number of DEXA scans increased significantly, with 47.7 in 2020, 57.0 in 2021, and 90.8 in 2022. The DEXA rate among proximal femoral fracture patients increased from 23.3% in 2020 to 88.1% in 2021 and 100% in 2022. The rate of treatment initiation also increased remarkably, from 21.7% in 2020, to 68.7% in 2021, reaching 97.8% in 2022. We performed 504 interventions, resulting in 251 patients diagnosed with osteoporosis, of whom 134 (56 from non-orthopedic departments) successfully started medication.

**Conclusions:**

Our innovative FLS system, incorporating an admission screening form and osteoporosis educational appointments, proved effective in identifying patients with osteoporosis and facilitating medication initiation, which will prevent both primary and secondary fractures.

## Introduction

Population aging is a global problem, with aging rates over 65 years old reaching 29.8% in Japan, 22.2% in Germany, 21.3% in France, 18.9% in the United Kingdom, and 16.7% in the United States in 2021 [1]. In Yaita City, the location of our hospital, the population aging rate is 33.9%, exceeding the national average [2].

Healthy life expectancy, the number of disease- and disability-free years an individual can expect to live, is a key measure used by the World Health Organization (WHO) to assess the health and well-being of a nation. The average life expectancy for Japanese individuals was 81.0 years for men and 87.1 years for women in 2016. However, the difference between average life expectancy and healthy life expectancy was 8.9 years for men and 12.3 years for women [3]. This disparity indicates a decline in the quality of life, increased burden on families, and increased social security costs, including medical expenses and long-term care benefits. A concerted effort to extend healthy life expectancy is needed to bring it closer to average life expectancy.

According to a survey of support and nursing care requirements in Japan, musculoskeletal problems account for one-fifth of cases, with 12.5% attributed to fractures and falls and 10.8% to joint diseases [4]. Osteoporotic fractures can result in immobilization, chronic back pain, and long-term care dependency.

Osteoporosis is described by WHO as a condition typified by reduced bone mass, abnormal bone tissue microstructure, increased bone fragility, and elevated fracture risk.

In 1994, WHO published a report focusing on fracture risk assessment in postmenopausal osteoporosis screening [5]. The report outlined diagnostic criteria based on measurements of bone mineral density (BMD) and acknowledged osteoporosis affected over 75 million individuals across the USA, Europe, and Japan.

Although the Japanese population is approximately 125 million and is unchanged, the number of osteoporosis patients in Japan continues to rise annually and is estimated to have reached 12.8 million individuals in 2015 (3 and 9.8 million men and women, respectively) [6]. Consequently, fracture prevention through osteoporosis intervention has become a pressing concern. Underscoring the importance of secondary fracture prevention, DM Black et al. demonstrated that after a vertebral fracture, the likelihood of subsequent vertebral fractures increases by a factor of 4.7, and the probability of hip fractures by 2.6 [7].

The fracture liaison service (FLS), initially established in the United Kingdom in the late 1990s, has expanded worldwide [8]. FLS provides secondary prevention for fragility fractures by involving various medical professionals. By systematically and proactively identifying patients who have sustained fragility fractures and assessing their risk of future fragility fractures, FLS offers guidance and therapies to mitigate the risks. Evidence indicates that this service increases BMD testing, facilitates the initiation of appropriate medication, and reduces the incidence of fragility fractures.

In Japan, the Osteoporosis Liaison Service (OLS) is an initiative to prevent fragility fractures associated with osteoporosis [9]. OLS is a unique Japanese system developed by the Japan Osteoporosis Society that targets wide range of patients with osteoporosis and older people, and is intended to prevent primary fractures via cooperation of physicians and medical staff of various professions, led by the osteoporosis manager certified by the Japan Osteoporosis Society. Osteoporosis manager certification is granted to those with national qualifications such as nurses, radiology technicians, clinical laboratory technicians, physical therapists, occupational therapists, pharmacists, and dietitians, who have attended specific lectures and passed an examination and obtained certification. In total, 3928 people had been certified as osteoporosis manager until April 2023, and of these, 48% were nurses; 19%, physical therapists; 16%, pharmacists; 6%, radiology technicians; 3%, dietitians; 2%, occupational therapists; and 6%, others. Recognizing the importance of secondary fracture prevention, particularly in patients with fragility fractures, the Japanese Osteoporosis Society and Fragility Fracture Network–Japan (FFN-J) published the Japanese version of the FLS clinical standards in 2019 [10] outlining the minimum indicators for secondary fracture prevention. There are five stages: identification, investigation, initiation, integration, and information.

In 2022, the medical service reimbursement introduced a new evaluation criterion for patients with hip fractures undergoing osteoporosis evaluation and treatment, aligned with guidelines such as those from the FLS and OLS. This reflects the growing recognition and adoption of FLS and OLS in Japan.

**Fig. 1:**
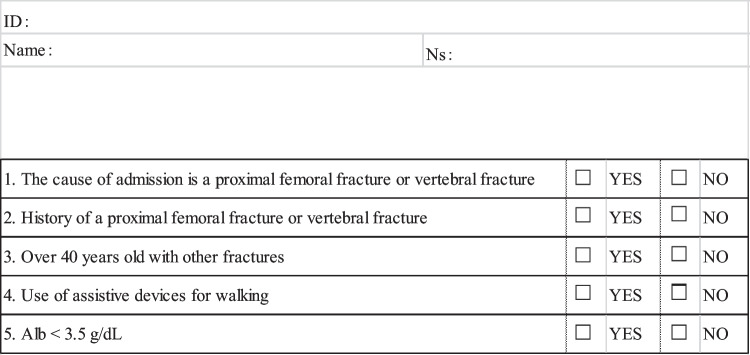
Admission screening form for osteoporosis. If one of the answers is yes, a request for team intervention is issued

Our FLS team established two primary goals. The first was ensuring that patients admitted to the orthopedic department, especially those with proximal femoral fractures, undergo comprehensive evaluation and receive appropriate medication. The second goal entailed identifying individuals with osteoporosis admitted to all departments, and initiating outpatient medication and care following discharge. The FLS team devised a novel system to screen all hospital admissions using a distinctive admission screening form (Fig. 1) and scheduling osteoporosis educational appointments with rehabilitation staff.

Our aim is to diminish the number of fracture cases within the community by identifying osteoporosis patients and enhancing treatment initiation rates. This study aimed to demonstrate the impact of our FLS team’s interventions on the frequency of DEXA scanning, treatment initiation rates, and the identification of osteoporosis patients among all hospitalized individuals.

## Methods

### FLS intervention

Our hospital’s FLS began operating in April 2022. The FLS team comprises a physician, outpatient and acute ward nurses, recovery ward staff, regional cooperation staff, a pharmacist, a radiologist, physical therapists, occupational therapists, nutritionists, medical affairs personnel, and care managers, all providing comprehensive support. The procedures followed at each stage are outlined below.

#### Stage 1: Identification

- All hospitalized patients are screened using a designated form (Fig. 1)
  - Admission cause: proximal femoral fracture or vertebral fracture
  - History of proximal femoral fracture or vertebral fracture
  - Age over 40 with other fractures
  - Use of assistive devices for walking (indicating fall risk)
  - Albumin level < 3.5 g/dl (indicating malnutrition)

For fresh lumbar spine compression fractures, MRI is finally performed for making a diagnosis. Regarding old lumbar spine compression fractures, the decision is based on the patient’s own or family’s report. In accordance with previous studies, the reference value for malnutrition was set at 3.5 g/dL albumin [11]. If any criteria apply, the patient is at high risk of fracture, and an intervention request is forwarded to the FLS team.

#### Stage 2: Investigation

- DEXA measurement of BMD
- Blood test to evaluate bone metabolism markers
- Fall risk assessment conducted by rehabilitation staff

#### Stage 3: Initiation

- Orthopedic patients
  - Initiation of medication during hospitalization
- Non-orthopedic patients
  - Diagnosis occurs during hospitalization
  - Provision of printed instructions emphasizing the importance of osteoporosis treatment upon discharge
  - Osteoporosis educational appointment scheduled on the same day as the outpatient visit following discharge, during which an osteoporosis manager provides a detailed explanation of treatment importance
  - Referral to an orthopedic specialist to initiate medication

#### Stage 4: Integration

- Yearly appointments for DEXA scans
- Facilitation of continued medication through regional cooperation with nearby clinics
- Telephone follow-up with patients who have not been seen

#### Stage 5: Information

- Regular FLS team workshops
- Attendance at sponsored lectures by pharmaceutical companies
- Seminars for all employees

**Fig. 2:**
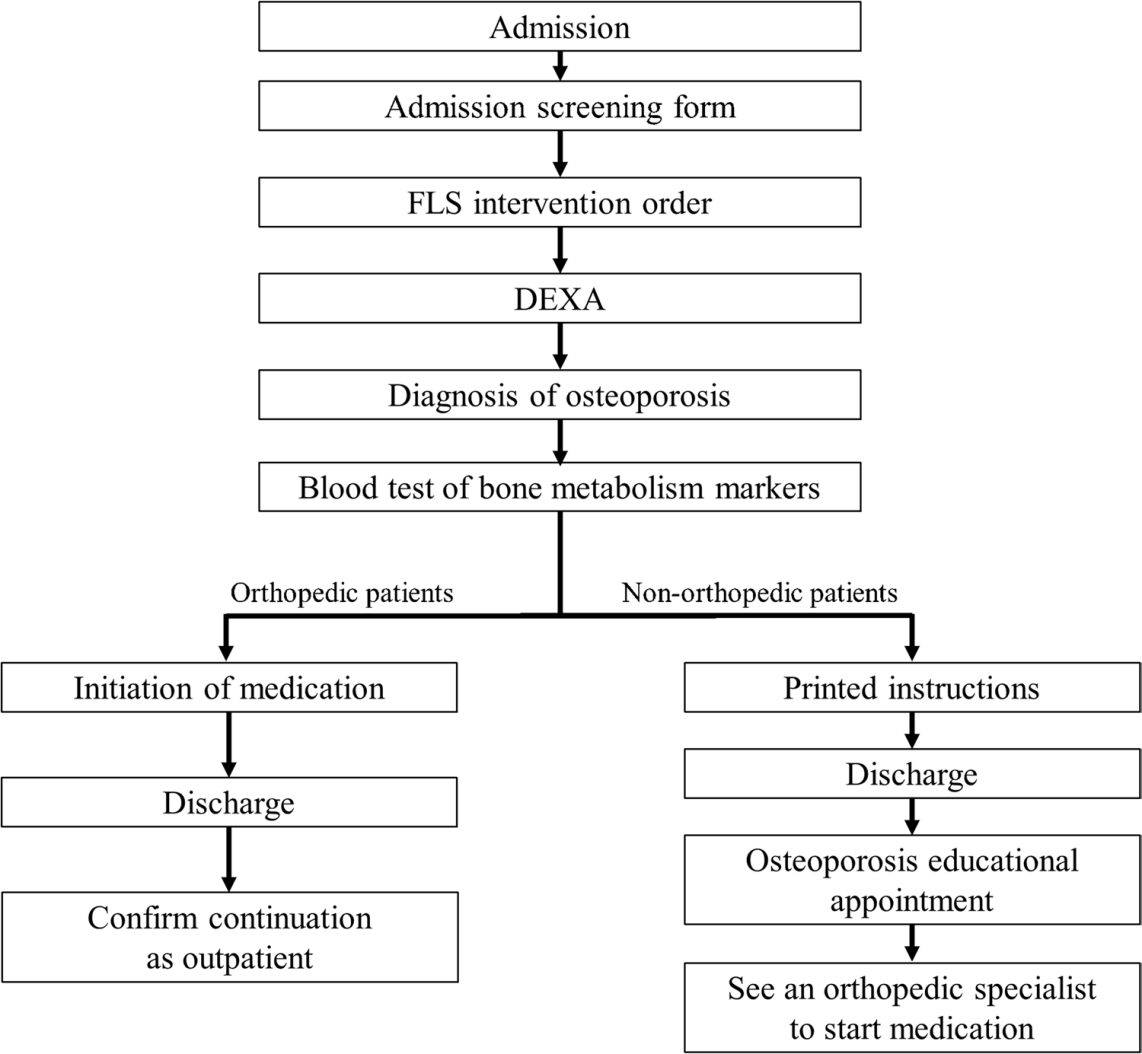
FLS intervention flow

The intervention process for inpatients is depicted in Fig. 2. All patients complete the admission screening form. FLS intervention is requested for patients identified as having a high risk of fracture. The patients have a BMD test, and if osteoporosis is confirmed, blood tests are conducted to assess bone metabolism markers. In addition to general items such as electrolytes, renal, and liver functions, bone metabolism markers such as P1NP, bone-specific alkaline phosphatase (BAP), and TRACP-5b were tested. Orthopedic patients begin medication immediately, while non-orthopedic patients are diagnosed and provided with printed instructions highlighting the importance of osteoporosis treatment upon discharge. Non-orthopedic patients attend an osteoporosis educational appointment on the same day as their outpatient visit following discharge. A certified osteoporosis manager from the rehabilitation staff, discussed inpatient DEXA results, general knowledge of osteoporosis, fracture risk, the necessity of osteoporosis treatment, and exercise therapy. If the patient consents, an outpatient orthopedic appointment is scheduled. This process ensures intervention for orthopedic and non-orthopedic patients with osteoporosis. Patients in the non-orthopedic group vary widely, including cardiology patients with heart failure and atrial fibrillation, patients on glycemic control admitted to the Division of Diabetes and Metabolism, patients with stroke admitted to the Division of Neurosurgery, and patients with metabolic encephalopathies admitted to the Division of Neurology and likewise. The drugs available in our hospital included bisphosphonates, teriparatide, denosumab, romosozumab, raloxifene, and vitamin D.

## Data analysis

### Capture the proximal femoral fractures

We summarized the total number of DEXA examinations, the DEXA examination rate for patients with proximal femoral fractures, and the medication initiation rate for each year between April 2020 and March 2023. This measured our FLS team’s first objective of providing reliable intervention for proximal femoral fractures.

### Effect of admission screening form and osteoporosis educational appointment on identifying osteoporosis patients and facilitating medication initiation

We summarized the total number of FLS team interventions, bone density tests, diagnosed osteoporosis, and treatment initiated between April 2022 and March 2023. This measured the second objective of our FLS team, i.e., identifying patients with osteoporosis admitted to all departments and initiating outpatient medication and care following discharge.

## Results

### Capture the proximal femoral fractures

The results of this study are presented using fiscal years: April 2020 to March 2021, April 2021 to March 2022, and April 2022 to March 2023 are referred to as years 2020, 2021, and 2022, respectively.

**Fig. 3:**
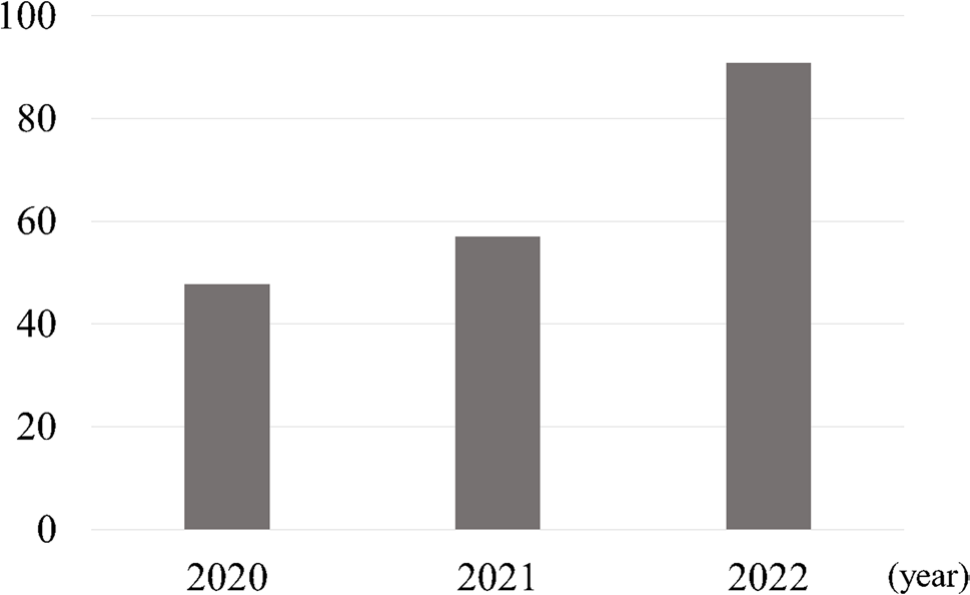
Number of DEXA scans (monthly average)

**Fig. 4:**
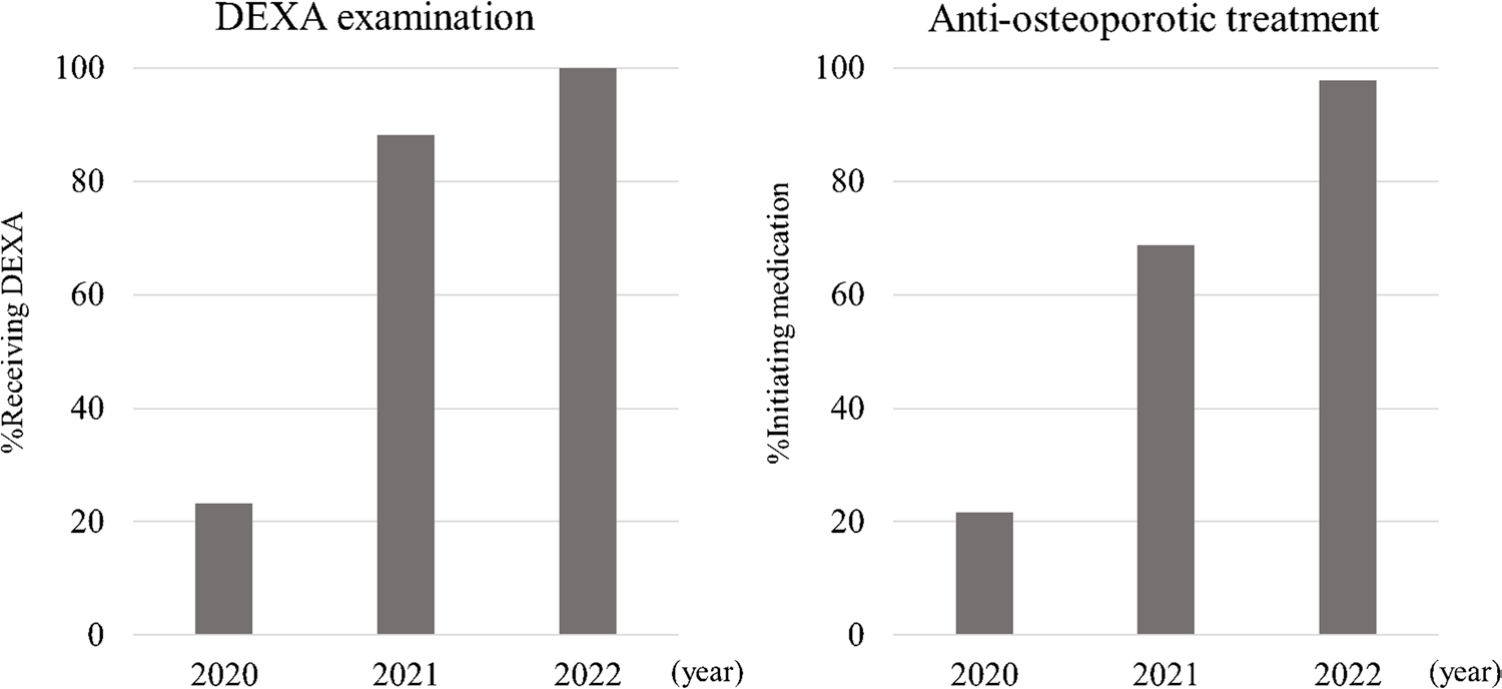
Intervention rates for patients with proximal femoral fractures

The average monthly number of DEXA examinations increased significantly, from 47.7 in 2020, to 57.0 in 2021, and 90.8 in 2022 (Fig. 3). The DEXA examination rate for patients with proximal femoral fractures increased from 23.3% in 2020 to 88.1% in 2021 and 100% in 2022. Similarly, the rate of treatment initiation rose remarkably from 21.7 to 68.7%, and 97.8% in 2020, 2021, and 2022, respectively (Fig. 4). All patients were started on bisphosphonates and vitamin D.

### Effect of admission screening form and osteoporosis educational appointment on identifying osteoporosis patients and facilitating medication initiation

**Fig. 5:**
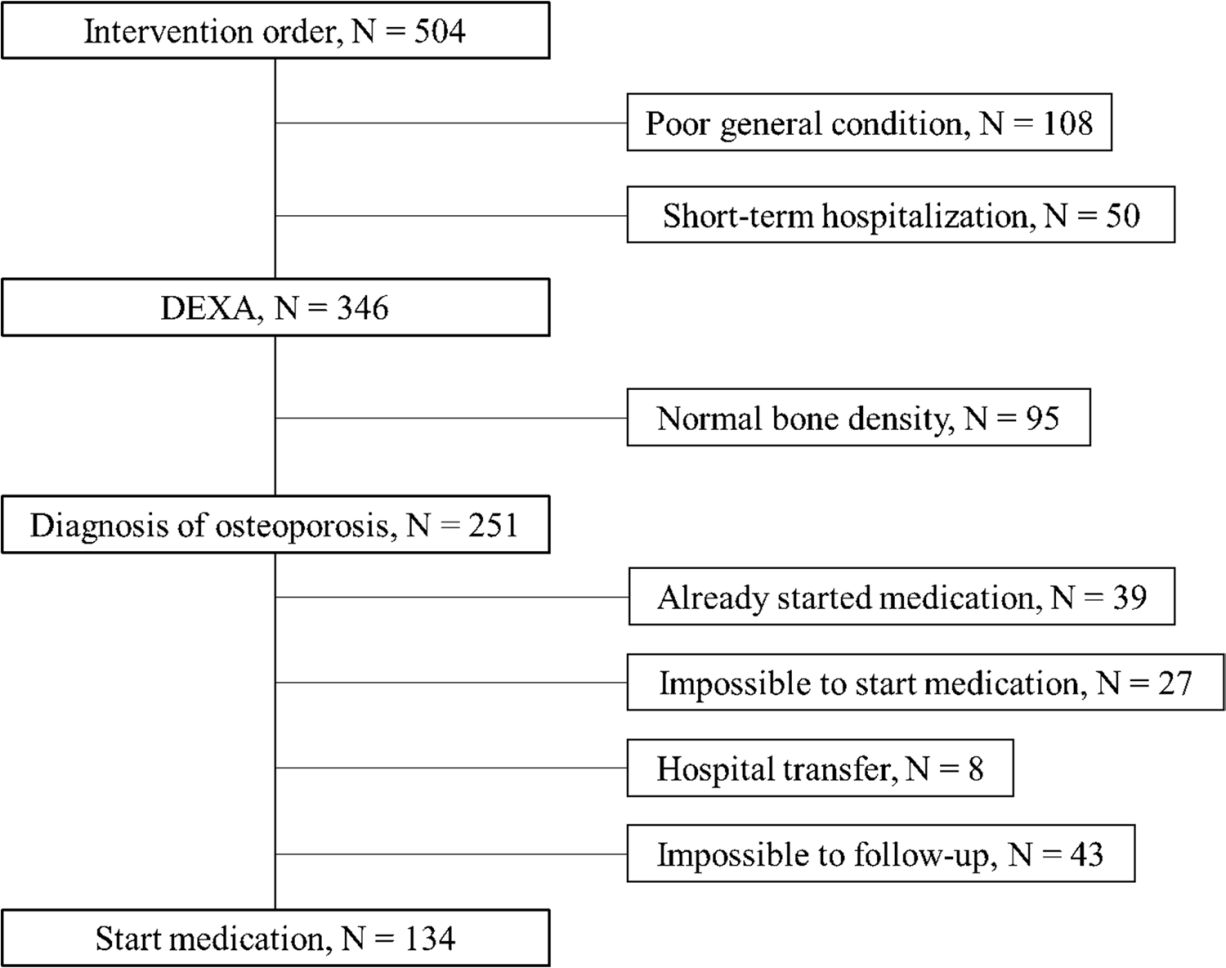
Participant selection flow chart

Regarding FLS team interventions, detailed results are presented in Fig. 5 and Table 1. The results of the responses to the admission screening form are shown in Table 2. In total, 109 patients answered yes to the question: The cause of admission is a proximal femoral fracture or a vertebral fracture. Forty-three patients answered yes to the question: History of proximal femoral fracture or a vertebral fracture. Forty-three patients answered yes to the question: Over 40 years old with other fractures. Three hundred nineteen patients answered yes to the question: Use of assistive devices for walking, and 208 patients answered yes to the question: Alb < 3.5 g/dL. Regarding the number of patients answering yes to multiple questions, two questions were 163; three questions were 23; four questions were 3; and five questions were 0.

**Table 1:** Details of the participants (orthopedics and non-orthopedics)

	Orthopedics	Non-orthopedics	All
Intervention order	131 (26.0%)	373 (74.0%)	504
DEXA	123 (35.5%)	223 (64.5%)	346
Diagnosis of osteoporosis	101 (40.2%)	150 (59.8%)	251
Start medication	78 (58.2%)	56 (41.8%)	134

**Table 2:** Results of admission screening form

Screening questions	Number of participants answering yes
1. The cause of admission is a proximal femoral fracture or vertebral fracture	109
2. History of a proximal femoral fracture or vertebral fracture	43
3. Over 40 years old with other fractures	43
4. Use of assistive devices for walking	319
5. Alb <3.5 g/dL	208
Multiple yes	
2 questions	163
3 questions	23
4 questions	3
5 questions	0

There were 504 intervention orders, with 131 (26.0%) for orthopedic and 373 (74.0%) for non-orthopedic patients. DEXA examinations were performed on 346 (68.6%) patients (123 orthopedic and 223 non-orthopedic), excluding 108 (21.4%) patients with poor general condition and 50 (9.9%) patients with short-term hospitalization (e.g., examination hospitalization, minor surgery, chemotherapy). Of the 346 patients who underwent DEXA, 251 (72.5%) (101 orthopedic and 150 non-orthopedic) were diagnosed with osteoporosis, while 95 (27.5%) patients had normal bone density. The details of 251 patients diagnosed with osteoporosis are shown in Table 3. No statistically significant differences were found in age and bone density.

**Table 3:** Details of the patients diagnosed with osteoporosis (orthopedics and non-orthopedics)

	All	Orthopedics	Non-orthopedics	*p*
Age	83.3 ± 8.8	82.9 ± 9.8	83.6 ± 8.1	0.58
Male	61	14	47	
Female	190	87	103	
BMD T-score of lumbar spine	−1.95 ± 1.66	−2.07 ± 1.43	−1.87 ± 1.81	0.39
BMD T-score of femoral neck	−3.06 ± 0.86	−3.17 ± 0.91	−2.99 ± 0.82	0.13

Of the 251 osteoporosis patients, 134 (53.3%) (78 orthopedic and 56 non-orthopedic patients) began medication. Details of the initiated anti-osteoporosis drugs are shown in Table 4. A total of 39 (15.5%) patients had already been introduced to anti-osteoporosis drugs, which included: 15 patients taking bisphosphonates (BP), 8 taking BP + vitamin D, 5 taking denosumab, 4 taking romosozumab, 3 taking teriparatide, 3 taking vitamin D, and 1 taking raloxifene. Of the 78 patients who did not receive treatment, 27 (34.6%) patients experienced challenges with medication administration (e.g., renal dysfunction, severe heart failure, electrolyte abnormalities, difficulties taking medication), 8 (10.3%) patients were transferred to other hospitals, and 43 (55.1%) patients were lost to follow-up. Nineteen patients with osteoporosis in the non-orthopedic group were seen for the osteoporosis educational appointment, 13 (68.4%) of whom were seen by an orthopedic specialist eventually leading to treatment.

**Table 4:** Details of the initiated anti-osteoporosis drugs (orthopedics and non-orthopedics)

	Orthopedics	Non-orthopedics
Bisphosphonates + vitamin D	55 (70.5%)	6 (10.7%)
Bisphosphonates	3 (3.8%)	23 (41.1%)
Teriparatide	10 (12.8%)	6 (10.7%)
Denosumab	2 (2.6%)	10 (17.9%)
Romosozumab	3 (3.8%)	7 (12.5%)
Raloxifene	1 (1.3%)	0 (0%)
Vitamin D	4 (5.1%)	4 (7.1%)
Total	78	56

## Discussion

Increases in examination and treatment initiation rates have already been reported with FLS intervention, and its impact on fracture prevention has also been documented [12–16]. The number needed to treat (NNT), a statistical measure of treatment efficiency, is 20 for fracture prevention with FLS intervention [17], in contrast to 119 for the prevention of coronary artery disease using hyperlipidemia medication, and 255 for myocardial infarction [18]. Several studies have demonstrated the cost-effectiveness of FLS [19–22] confirming it as a highly efficient intervention for fracture prevention.

Our hospital operates on approximately 60 proximal femoral fracture cases annually. FLS intervention has facilitated reliable testing and intervention for proximal femoral fractures. The level of interest in osteoporosis varies among orthopedic surgeons. Our team has two experienced doctors in leadership positions and two younger doctors. The younger doctors tend to prioritize surgical procedures over the prevention of secondary fractures. In 2020, doctors with a limited interest in prevention were involved in teaching, resulting in an examination rate of only 23.3% and a treatment initiation rate of 21.7%. Even considering the data from previous references, it is reasonable to assume that the examination and treatment initiation rates were around 20% [23, 24]. In 2021, after one of the authors of the present paper (SK) was assigned to teaching, the examination and treatment initiation rates significantly improved. With the support of the FLS team, we incorporated DEXA into the clinical pathway, achieving a 100% testing rate in 2022. The treatment initiation rate reached 97.8%, with only one patient refusing medication. The first FLS team objective was achieved.

The second objective was to identify and initiate treatment for osteoporosis patients admitted to other departments. In 1 year, the FLS team initiated medication for 56 non-orthopedic osteoporosis patients who would have previously been overlooked. Unlike conventional Japanese FLS teams that solely focus on patients with proximal femoral and vertebral fractures, our FLS team was innovative in identifying osteoporosis patients among all admissions [25].

Nevertheless, there were challenges regarding patients admitted in other departments. The first challenge was determining which patients should undergo DEXA for BMD testing. We developed a simple admission screening form aiming to keep the screening as straightforward as possible. Secondly, if the DEXA test revealed osteoporosis, the next challenge was deciding when to initiate medication. Orthopedic surgeons could not intervene aggressively in another department due to the patient’s overall health condition. Moreover, communication problems with doctors from other departments arose. It was also important to explain the diagnosis to the patient’s family members, who play a crucial role.

We decided to perform only the examination and diagnosis, with intervention after discharge. Initially, orthopedic outpatient appointments were scheduled simultaneously with the other appointments, but patients were often confused by the mixed instructions or refused to comply. We devised a strategy to provide osteoporosis education from a certified osteoporosis manager during the outpatient waiting time. Many patients had already received rehabilitation intervention during their hospital stay. They readily accepted guidance from already familiar rehabilitation staff and seamlessly transitioned to the orthopedic outpatient department. Many patients were satisfied with the detailed explanations obtained from osteoporosis managers. When patients visit the outpatient department, there is typically a waiting time for blood tests and other procedures, which can be stressful and unproductive. This system effectively uses that time. While simple and easy to implement, this system is novel and unique in Japan. It has enabled the identification and intervention of non-orthopedic patients with osteoporosis. Many patients were satisfied with the detailed explanations provided by the osteoporosis managers. Interestingly, about 68.4% of the patients who came to the osteoporosis educational appointment saw an orthopedic specialist; however, we need to raise this value. The current challenge is to conduct DEXA tests on relatively healthy patients hospitalized for short periods and to initiate medication for patients diagnosed with osteoporosis but lost to follow-up.

To efficiently examine and initiate osteoporosis treatment, it is crucial for the entire hospital to recognize the FLS intervention as a valuable endeavor for patients and the hospital. We operate efficiently by keeping the screening form as simple as possible and conducting meetings during work hours. Additionally, our hospital has six certified osteoporosis managers (three nurses, and one pharmacist, physical therapist, and occupational therapist) who actively engage in patient education. The staff are motivated to educate patients.

Our FLS team has only been operational for approximately 1 year, and therefore cannot yet assess fracture prevention rates. Nonetheless, we aim to maintain our high examination and treatment initiation rates, and sustain a high treatment continuation rate, which will ultimately contribute to long-term fracture prevention in the community.

In conclusion, our innovative FLS system, with admission screening and osteoporosis educational appointments, has proven effective in identifying osteoporosis patients and initiating medication. Further research on long-term FLS intervention is necessary to demonstrate its effectiveness in fracture prevention.

## Data Availability

Data sharing is not applicable to this article as no datasets were generated or analyzed during the current study.
